# Describing the impact of health research: a Research Impact Framework

**DOI:** 10.1186/1472-6963-6-134

**Published:** 2006-10-18

**Authors:** Shyama Kuruvilla, Nicholas Mays, Andrew Pleasant, Gill Walt

**Affiliations:** 1 Health Services Research Unit, Department of Public Health and Policy, London School of Hygiene and Tropical Medicine, London, UK; 2 Health Services Research Unit, Department of Public Health and Policy, London School of Hygiene and Tropical Medicine, London, UK; 3 Department of Human Ecology and Department of Family and Community Health Sciences, Rutgers University, New Brunswick, USA; 4 Department of Public Health and Policy, London School of Hygiene and Tropical Medicine, London, UK

## Abstract

**Background:**

Researchers are increasingly required to describe the impact of their work, e.g. in grant proposals, project reports, press releases and research assessment exercises. Specialised impact assessment studies can be difficult to replicate and may require resources and skills not available to individual researchers. Researchers are often hard-pressed to identify and describe research impacts and ad hoc accounts do not facilitate comparison across time or projects.

**Methods:**

The *Research Impact Framework *was developed by identifying potential areas of health research impact from the research impact assessment literature and based on research assessment criteria, for example, as set out by the UK Research Assessment Exercise panels. A prototype of the framework was used to guide an analysis of the impact of selected research projects at the London School of Hygiene and Tropical Medicine. Additional areas of impact were identified in the process and researchers also provided feedback on which descriptive categories they thought were useful and valid vis-à-vis the nature and impact of their work.

**Results:**

We identified four broad areas of impact:

I. Research-related impacts;

II. Policy impacts;

III. Service impacts: health and intersectoral and

IV. Societal impacts.

Within each of these areas, further descriptive categories were identified. For example, the nature of research impact on policy can be described using the following categorisation, put forward by Weiss:

*Instrumental use *where research findings drive policy-making;

*Mobilisation of support *where research provides support for policy proposals;

*Conceptual use *where research influences the concepts and language of policy deliberations and

*Redefining/wider influence *where research leads to rethinking and changing established practices and beliefs.

**Conclusion:**

Researchers, while initially sceptical, found that the Research Impact Framework provided prompts and descriptive categories that helped them systematically identify a range of specific and verifiable impacts related to their work (compared to ad hoc approaches they had previously used). The framework could also help researchers think through implementation strategies and identify unintended or harmful effects. The standardised structure of the framework facilitates comparison of research impacts across projects and time, which is useful from analytical, management and assessment perspectives.

## Background

Researchers are increasingly, and regularly, requested to describe the impact of their work in:

• Grant proposals, especially sections on research users, beneficiaries, communication and expected impact;

• Research project evaluations and reports, particularly to provide accountability for funds invested in research;

• Publicity information e.g. for institutional annual reports and press releases that highlight "success stories";

• Research dissemination and implementation strategies;

• Assessments of the impact of research on policy, practice and public opinion;

• Individual and institutional research assessment exercise (RAE) submissions.

However, researchers are often hard-pressed to come up with coherent and comprehensive narratives of actual or potential research impacts and produce ad hoc accounts that do not facilitate comparison across time or learning across cases. More specialised research impact assessment studies can be difficult to replicate and may require resources and skills not available to individual researchers [1,2]. Further, the specialised focus of these models, for example on publications and economic benefits, may not cover a range of other areas where research could have impact. For example with regards to health research, environmental effects and social capital are increasingly associated with health outcomes and are areas where research can have impact [3-5], but are often neglected in existing health research impact assessment models and frameworks. A research impact scoping exercise at the London School of Hygiene and Tropical Medicine (LSHTM) identified some additional areas in which health research can have impact, such as on policy networks and on other sectors such as energy, that are also often missing in existing frameworks and models of health research impact [6].

In this paper we take a practical approach to help researchers describe the impact of their work. We present the *Research Impact Framework*, which is designed as a 'DIY' approach with descriptive categories that prompt researchers to systematically think through and describe the impact of their work. The framework was also designed with some additional goals in mind: to develop a standardised framework to help describe impacts across research topics and methods and facilitate comparison across time and cases; to guide researchers in planning research implementation and evaluation strategies; to facilitate researchers in looking at the broader influences and effects on and of their work in society; to promote research accountability in relation to the use of resources and the consequences of research; to help in the attribution of effects to health research given the range of other determinants of health and societal impacts; and to contribute to more extensive or specialised evaluations of research impact.

Researchers' narratives of the impact of their work could also serve as building blocks for a variety of more specialised analytical purposes. For example, researchers' accounts are considered a starting point for more specialised research impact assessments and can provide similar findings, though the level of detail and analysis may differ [7]. In analysing the role of research in diffusion of innovations, Greenhalgh et al. [8] found that "researchers in different traditions had conceptualised, explained and investigated diffusion of innovations differently and had used different criteria for judging the quality of empirical work. Moreover, they told very different over-arching stories of the progress of their research." Thus researchers' narratives also facilitate analysis of differences in the 'doing', diffusion and impact of research within and across research fields [8].

While the Research Impact Framework provides a simple, practical approach to developing research impact narratives, it is important to recognise that these narratives are generated and assessed in the context of historically rich and complex, often contending, views on the role of science and its relationship with society. Some may align with Bacon's [9] utopian vision wherein wise and benevolent rulers coordinate and disseminate research for the good of the state. Others take a 'Republic of Science' view, espoused by Polyani [10] and Vannevar Bush [11], where science is idealised as an independent enterprise, separate from societal concerns and having intrinsic value in and of itself. However, researchers on the sociology of scientific knowledge posit that science and society are interlinked and mutually influential [12,13]. Further, Kuhn [14] and Callon [15] among others, discuss how science itself is a social enterprise with researchers interacting within specific scientific cultures and communities.

There may also be different types of impact expected from different types of research, for example basic, applied, action, clinical, user driven, translational or curiosity-driven research. Then there are contesting and complementary theories and models of causal pathways of research impacts associated with different levels and types of impact on research, policy and practice [16-19]. Several academic disciplines including communication, diffusion of innovation studies, policy science, sociology of scientific knowledge and organisational research all offer valuable perspectives on different aspects of assessing the production, communication, utilisation and impact of research [20]

Further considerations in describing research impacts include questions of accountability, for example, whether there is a different standard of accountability for researchers compared with practitioners with regards to impact [21]. There are also concerns about whether research impacts are positive or negative (and for whom) and what individual or institutional biases and incentives may operate in describing impact. Prioritisation of impact is another assessment issue, though priorities may vary in accordance with different funders' priorities, in different research fields and in different socio-political contexts.

Researchers may variously ascribe to different worldviews and may take into account various assessment considerations, either knowingly or unknowingly, when describing the impact of their work. However, the Research Impact Framework presented in this paper is not aligned with any particular philosophy, is not in itself evaluative and does not prioritise impacts or propose causal pathways. The Research Impact Framework was primarily designed as a practical tool to help researchers think through and describe the impact of their work; this could then serve a range of practical and analytical purposes as required.

While much of the framework presented in this paper could apply to research impact narratives in general, specific considerations warrant a topical focus on health research, which is defined as "the generation of new knowledge using the scientific method to identify and deal with health problems" [22]. While health research is nested within the larger science and technology enterprise, there are a multitude of social, governmental, academic, service, manufacturing and legal institutions specifically in place to deliver the products of health research to society [23]. Further, a systematic review on the diffusion of innovations, including research, emphasized the need for "building up a rich picture of process and impact" in health services [24].

To address the gaps in existing approaches to and models of health research impact assessments, we first drew from the literature on health research impact assessment models and criteria to map out a framework of health research impacts. This framework was then tested and modified against impacts identified by LSHTM researchers working on a wide range of health research topics. Using the Research Impact Framework allowed individual researchers to identify and select impacts relevant to their work and develop impact narratives without requiring specialised skill in the field of research impact assessment. The standardised descriptive categories also facilitated analysis across the narratives. The methods used to develop the Research Impact Framework are described in the following section.

## Methods

Our objective was to develop a conceptual framework to help health researchers think through and describe the possible outcomes of their work. A framework is a way of setting out a range of possible variables related to the issue of interest, but does not necessarily identify the relationships between them [25] or provide an evaluative judgement of the variables. For example, the fallout from the controversy of research linking the MMR vaccine with autism received substantial coverage in scientific journals and in the media and had various adverse effects such as a drop in immunisation rates [21]; a framework would include these areas of impact, but would not evaluate the nature of this impact. We developed a conceptual framework that covered a wide range of potential areas of health research impact, and standardised ways of describing them, so that individual researchers without any specific training in research impact assessment could use the framework to describe the impact of their work, selecting descriptive categories as per relevant purposes, priorities and assessment criteria.

To develop the Research Impact Framework, we first mapped out potential research impact areas based on a review of the main health research impact assessment approaches. The goal was not to conduct an exhaustive review of the literature, or to analyse the causal claims of different impact assessment models, but only to identify and map out the scope and coverage of potential areas of health research impact across a range of models, frameworks and criteria. The approaches we drew on to map the health research impact areas, and there were was overlap between them, were the Payback Model of health research benefits [18], a framework to analyse Health Research Systems [23], a 'knowledge transfer' approach to assessing the impact of research [26], a model of the path from evidence generation to clinical application [27] as well as economic approaches to assessing health research impact, such as the Funding First approach, which includes calculations of the economic value of increased life expectancy resulting from investments in research [28]. We also drew on frameworks that did not explicitly focus on health research, but explicated the links between globalization and health [3], pathways of communication and social change [29] outcomes of health promotion [30] and the non-financial constraints in health systems [31] as these are all areas where health research potentially could have impact. Research assessment criteria, for example, as proposed in the OECD's Oslo Manual [32], criteria for the initiation and evaluation of National Institutes for Health (NIH) Extramural Center Programs [33] and the UK Research Assessment Exercise [34], further informed the development of the Research Impact Framework by highlighting contemporaneous criteria against which the significance and impact of research are judged.

We turned this initial mapping of potential research impact areas into a semi-structured interview guide, designed to be used in interviews with researchers to develop narratives, or case studies of research impact (See Table 1). LSHTM-based projects were purposefully selected for maximum variation [35] with reference to project topics and with regards to the familiarity of the principal investigators with research impact assessment concepts. The study was initially developed in the Department of Public Health and Policy (PHP) at LSHTM and included primary analysis of the impact of seven research health services and policy research projects and secondary analysis of four other research projects based on impact assessments previously conducted [6]. These projects covered a wide range of topics: health care financing reform, bilingual young people's agency in facilitating health care access for their families, climate change and health, primary care-led commissioning in the NHS, a clinical audit of sino-nasal surgery, epidemiological analysis of cold-related morbidity and mortality, the public health implications of the trafficking of women and adolescents, the UK National Prospective Tonsillectomy Audit and HIV/AIDS-related research [6]. The study was then expanded beyond PHP to further develop the Research Impact Framework based on analysis of four 'basic' or 'technical' research projects selected from the departments of Epidemiology and Public Health (EPH) and Infectious and Tropical Disease (ITD). These research projects focused on insecticide treated bed nets to prevent malaria, handwashing with soap to prevent diarrhoeal diseases, morbidity and mortality related to road traffic injuries, and the use of epidemiological methods in genomics research.

The principal investigators (or LSHTM focal points in collaborative projects) of the selected research projects were interviewed using the semi-structured interview guide that was structured according to the mapping of research impact areas (See Table 1). Principal investigators were selected as the main interviewees because they were primarily responsible for drawing up descriptions of research impact in grant proposals as well as in final project reports. In addition, LSHTM colleagues whose expertise included research impact assessment as well as the heads of the research units were interviewed, not on the substance of the individual projects per se, but on the development of the Research Impact Framework. One investigator conducted all the interviews that took around 45 minutes each, and took contemporaneous notes. Information was also gathered from research project documents including reports, published papers and correspondence. The data were analysed based on a thematic analysis using the descriptive categories of the framework as the main themes.

This process helped to both test and further develop the framework, for example, through the identification of additional areas of impact, such as intersectoral services related to vector control, road safety and climate change, that were not included in the initial mapping based on the literature review alone. These data were used to further develop and refine the initial mapping of health research impact areas to produce a final version of the Research Impact Framework (See Table 2).

Within each impact area in the Research Impact Framework, we also identified descriptive categories in the literature, discussed in following sections, to support and help standardise the development of research impact narratives to enable comparative analysis across projects. Through an iterative and consultative process with LSHTM researchers involved in the selected projects, we determined which terms and categories were most useful to help researchers structure narratives of research impact.

The Research Impact Framework was thus validated through congruence with the literature on health research impact assessment, as well as through empirical analysis of research projects. Researchers in the LSHTM study found the approach more systematic than the more usual ad hoc and guess work-based process. The framework also provided new perspectives on potential and actual health research impacts and allowed for comparison of impact and assessment-related issues across the selected projects [6].

In this paper, the Research Impact Framework is presented, including the main health research impact areas and the descriptive categories within them. Examples from the LSHTM study and from the research impact assessment literature are used to illustrate key points.

## Results: the Research Impact Framework

We identified four broad areas to structure a framework of health research impacts, based on a literature review as well as an empirical analysis of selected LSHTM research projects:

I. Research-related impacts

II. Policy impacts

III. Services impacts: health and intersectoral

IV. Societal impacts

Within each of these areas, key descriptive categories were identified (See Table 2). By listing this broad range of health research impact areas, we do not propose that all these impacts should be expected or targeted all the time. Neither do we suggest that these are necessarily mutually exclusive categories. Rather, the framework offers an overview of potential, and sometimes overlapping, research impact areas that can serve as a series of prompts or a checklist that researchers can select from or modify, as appropriate to the research being described and taking into account, for example, funders' priorities or research assessment criteria.

## I. Research-related impacts

Research can have impacts within the research field itself that can be described using the following categories:

• *Type of problem/knowledge*

• *Research methods*

• *Publications and papers*

• *Products, patents and translatability potential*

• *Research networks *

• Leadership and awards

• *Research management*

• *Communication*

### Type of problem/knowledge addressed

The type of problems addressed and knowledge generated through research are primary areas in which to describe impact. The following adaptation of a categorization by Nutley, Walter and Davies [16] helps think through this area of research impact and research projects may address more than one of these categories.

• *Data about a problem/phenomenon: *e.g. statistics and trends showing declining vulnerability to temperature-related mortality in London over the 20th century [36].

• *Definitions and concepts: *e.g. the concept of equity in health care financing.

• *Rationale for action/possible solutions: *e.g. rationale for health promotion strategies based on evidence that hand washing with soap helps prevent diarrhoeal diseases [37].

• *Evidence of effectiveness of interventions: *e.g. through clinical audits of different surgical techniques.

• *Information on how to implement solutions: *e.g. methods to promote the purchase and treatment of bed nets with insecticide by communities themselves, an effective, equitable and sustainable strategy to prevent malaria [38-40].

• *New research topics in a field: *e.g. public health research has been extended to topics such as the public health implications of climate change [41] or road traffic injuries [42] and new knowledge on the determinants of health is continually produced.

• *Addressing research gaps and testing new hypotheses: *e.g. gaps in existing research can be identified through systematic reviews of the literature on a topic and studies may be designed to address these gaps or to test new hypotheses.

• *Ethical debates and guidelines: *e.g. the development of new ethical guidelines for research on violence against women [43].

• *Responsiveness/public interest: *e.g. research responding to topics of public interest or on government and media agendas, such as research on obesity or teenage pregnancy; or research that contributes to issues becoming topical in the media or in policy and analysis of the same.

### Research methods

This area of impact covers the mode of research employed or a researcher's methodological contribution to the overall study. Knowledge evolves along a continuum that ranges from applying models that have worked elsewhere to trying out something completely new [16]. Thus, in addition to describing the actual methods used (e.g. survey research, face-to-face interviews, case studies, multi-variate analysis) categories to describe methodological impact include:

• *Replication *of a study.

• *Application *of established methods.

• *Further development*/*extension *of methods, such as the application of Bayesian modeling techniques in emerging genomics research to study genotype-phenotype associations around a causative site, including relative risk of a causative allele and mode of inheritance [44].

• *Innovation *in terms of developing new research methods, for example, new methods for accessing "hard-to-reach" groups, and the use of new technologies such as GIS in research.

• *Synthesis *of research involves review and appraisal across several research projects and can help determine whether theories are adequately tested before applying them to practice and policy, indicates if resources are being wasted on new research on topics previously and conclusively studied, facilitates analysis of new evidence in the context of previous evidence, and highlights gaps in current knowledge. There are a variety of methods used to synthesise diverse forms of qualitative and quantitative evidence, for example systematic reviews as set out by the Cochrane [45] and Campbell Collaborations [46]; methodological development in the area of research synthesis is ongoing [47].

### Publications and papers

This is the perhaps most familiar part of the framework for researchers, as the impact of research is commonly described based on publications, in tenure and performance reviews to research utilisation analysis and research assessment exercised, using measures such as [33,34,48]:

• *Publications in scientific journals *and the 'impact factors' of the journals

• *Technical reports*, project reports, position statements etc, which while often considered as 'grey literature', can sometimes be more rigorously peer-reviewed than scientific publications and are widely read.

• *Citations *of research publications by other researchers.

### Products, patents and translatability potential

• *Products and processes*. The OECD Oslo Manual [32] classifies technological innovation as comprising:

◦ Introduction of a new product

◦ Qualitative change in an existing product, for example improved technologies, sometimes referred to as 'better mousetrap' solutions

◦ Process innovation that is new to an industry

◦ Opening of a new market

◦ Development of new sources of supply for raw materials or other inputs; and changes in industrial organisation.

Other specific examples of research products include vaccines and drugs, decision algorithms, mathematical models, information tools and resources e.g. geographical information systems; training manuals, databases, health education materials etc.

• *Patents *of research information or products and *citations *of patents by other researchers, are another area of potential research impact [32]. For example, an analysis of citations of U.S. industry patents showed a strong national linkage with "each country's inventors preferentially citing papers authored in their own country, by a factor of between two and four" [49].

• *Commercial development *of scientifically developed products including commercial licences and spin off companies. This may also include the influence of research on product development and marketing processes [32].

• *Translatability potential *of research refers to the potential for the translation of research findings, particularly in basic science, to clinical applications [33] or to technological opportunities and outcomes [32]. Indicators of translatability potential include publications in clinically oriented journals, patent applications, licenses issued and clinical trials underway or completed [33]. The term is also used to discuss the extent to which the research can be applied in other study contexts as well as in other disciplinary fields. This area of research impact is receiving increased attention as new, and at times controversial, partnerships between industry and business, government and non-governmental organizations and university-based research units and researchers continue to develop [50].

### Research networks and user involvement

Developing and maintaining research collaborations as well as client and user involvement in research are increasingly desired goals as they are seen to increase the reach, responsiveness, relevance, dissemination and impact of research [19,51,52]. Research partnerships usually involve researchers, research groups and institutes who jointly submit a research grant application, for example, and work together on a research project. Other partners as well as potential research clients and users may be involved at various stages of the research process from ethics review processes to research commissioning, collaborative research and project evaluation. For example, the Canadian Health Services Research Foundation specifically focuses on building 'linkage and exchange' between researchers and policy-makers in research priority setting, funding, proposal assessment and in the conduct and communication of research, in order to enhance the utilisation and impact of research on policy as well as to evaluate this impact [53]. Listing research collaborators, other partners, clients and users, as well as their specific involvement in the research, provides a useful description of this area of research impact.

### Research leadership

Leadership in research, is often a key area of interest in research assessment exercises [34] and may be related to a specific project or to a body of work. Research leadership can be documented by referring to:

• *Role in setting the agenda or standards *for a field of research or setting out a vision for the same.

• *Leadership in coordinating and managing *research projects and multi-institutional research collaborations.

• *Public recognition *of leadership including prestigious fellowships, named lectures and keynote addresses, awards that mark significant achievement in research, or membership in honorary scientific societies.

• *Membership of regional, national or international research bodies*, review boards and funding bodies.

• *Editorship of journals *or membership on journal editorial boards and advisory committees.

### Research system management

A health research system is defined as the people, institutions, networks and activities whose primary purpose is to generate, communicate and promote the utilization of scientifically validated knowledge that can be used to enhance, restore and/or maintain the health status of populations [23]. Research can have an impact on research system management by:

• *Expanding health research system linkages *for multidisciplinary and cross-sectoral research [33]. For example, the advantages of coordinating health and environmental impact assessments are increasingly recognized [54].

• *Changing research priority setting, investment strategies, resource allocation and accounting processes*. Research can highlight gaps in resource allocation for research, for example in relation to health research priorities and in terms of equitable allocation of research resources to address the needs of different populations [33,55].

• *Developing capacities to conduct research *and providing opportunities for training and development of researchers [23,33,56].

• *Changing the research environment *in relation to working conditions, incentives, job retention rates, recruiting and retaining women in science and overall researcher satisfaction [23,33].

• *Influencing health research system performance *and assessing comparative advantages of different research systems [23,32].

### Communication

Studies have long shown that researchers feel handicapped in their involvement in research communication due to lack of time, resources and incentives and that research is disseminated mainly through existing networks within the scientific community [52]. This is not only due to limited capacity, but also because the 'popularisation' of research has been perceived to be in conflict with the norms and standards of science [57]. These findings were reflected in the LSHTM study as well [6]. Nevertheless, as found in the LSHTM study, researchers do employ various modes of research dissemination, often on their own initiative. Potential target audiences and modes of dissemination for research are listed below.

Target audiences

• Academic/research institutions and programmes

• Donor government/bilateral agencies (e.g. DFID, USAID)

• Public/consumer organisations and groups

• Government offices/agencies

• Health insurance organizations

• Hospitals and clinics, practising physicians, nurses, allied health professionals etc.

• International organisations (e.g. UN, WHO, World Bank)

• Media and entertainment groups

• Non-governmental organisations (NGOs)

• Other private sector companies/institutes

• Patients

• Pharmaceutical companies

• Professional associations

• Research funding councils/foundations/charities

• Other research networks e.g. with more informal structures, sometimes termed epistemic communities [58]

**Modes of research dissemination and implementation strategies**[16,59]

• Audit and feedback processes

• Books: textbooks; technical; 'popular'

• Conferences and workshops

• Decision aids/algorithms/computer reminder systems

• E-mail/list serve discussions

• Face-to-face interaction/meetings

• Films, documentaries

• Guidelines

• Health education and health promotion

• Incentive systems (financial/professional) for use of research products

• Interventions targeting health that apply research findings, for example social marketing of soap to promote hand washing and prevent diarrhoeal disease

• Mass media: press releases and newspapers – articles and op eds; television; radio; magazine; and Internet

• Opinion leaders

• Organisational/systems-level strategies

• Policy briefs/recommendations

• Public engagement activities

• Formal academic talks/presentations

• Translation of research for different users

Beyond listing of research communication activities, specific impacts of research communication could also be usefully described [29], for example in curricular use or media coverage as described below.

#### Curricular/educational use

The use of research findings and papers in educational curricula and courses is a key area of research impact [19]. In the LSHTM study, researchers noted as impacts in this area feedback from other researchers and institutions about the inclusion of publications on the selected research projects in course curricula or being invited as guest speakers on courses.

#### Media coverage

Research effects relating to media coverage include [60,61]:

• Media coverage on a research project's findings; descriptive details including dates and any attribution or mention of a particular research project or researcher.

• The nature of the coverage in terms of scientific accuracy and whether the research was viewed favourable or unfavourably; e.g. using headlines or quotes to illustrate the nature of media coverage.

• Media coverage of individual researchers, e.g. their work, profile or personality; including whether researchers are depicted as being average everyday individuals or somehow different than the social norm; i.e. whether scientists are considered part of society or separate from it.

• The long-term trends in covering health research in the mass media; e.g. the extent individual researchers are used as sources of information in the media, the position of health research within the news agenda; and the representation of health research and researchers in entertainment content.

## II. Policy impacts

Increasingly, one of the main objectives of health research is to inform and influence policy and there are several potential impacts in this area:

• *Level of policy-making*

• *Type of policy*

• *Nature of policy impact*

• *Policy networks*

• *Political capital*

### Level of policy-making

Research can have an impact on policy-making at different levels and including different groups, for example, national and local politicians, health services administrators and managers/directors, representatives of local, national and international professional groups, NGOs and business leaders. In describing impact, the geopolitical level of policy impact, i.e. whether at international, national, or sub-national level, can be described as well as if the changes were localised to a particular organisation or network of organisations within those levels.

### Type of policy

The type of policy influenced by research is another way that impact can be described. For example, Black [62] endorses a distinction made between: practice policies, service policies and governance policies. Type of policies may also be described in relation to particular types of policy institutions, for example, researchers in the LSHTM study discussed how findings on home temperature-related morbidity and mortality [63] were used in the UK parliament in support of the Housing Bill (2004). The publication of WHO guidelines on ethics related to research on women and adolescents who had been trafficked [43] that had been developed on the basis of a research project was cited another example of policy impact in the LSHTM study [6].

### Nature of policy influence

There are several ways in which research can influence policy and Weiss (1998) identified four main modes of influence listed below [64]. There are a wide range of other categorisations of research to policy impact [65], including in Weiss's earlier work [17], describing rational, tactical and symbolic use of research in policy-making. In the LSHTM study, the following categories were found to be conceptually clear and relevant to policy impacts identified by researchers:

• *Instrumental use *where research findings directly drive or define policy.

• *Mobilisation of support *[supportive evidence] where research findings provide persuasive evidence to back ongoing and proposed policy activities or raise awareness and support for new policy-making, for example through writing policy briefs. As one LSHTM researcher noted, "Writing policy briefs gives us the opportunity to discuss the wider policy implications of our research and to anticipate and rehearse policy arguments ... Our work adds weight to policy deliberations." [6]

• *Conceptual use *where research leads to new ideas and language that influence the nature and substance of policy discourse, for example the concept of equity is increasingly used in considerations of health care financing [66,67].

• *Redefining/wider influence *refers to research impact that leads to a wide change or transformation of accepted beliefs and practices. This may involve overturning orthodoxies, as in the revolutionary shift in eighteenth century medical science from the concepts of vapours and humours to the constructs of anatomy and pathology [68]. More contemporary examples also indicate how research can lead to the changes in existing beliefs and practices. For instance, a multi-country randomised control trial found that widely accepted practice of administering corticosteroids after head injury could be harmful [69] with implications for both policy and practice on this topic.

### Policy networks

In relation to a specific policy problem, *networks *of actors and institutions with particular interests and perspectives on that issue may pre-exist or form and interact in policy-making [70]. At times, these networks are referred to as epistemic communities and extend across normally disparate elements of research, policy, practice and the public, but are based on a common research-based understanding of the issue [63]. As with the dissemination of research through research networks, policy ideas and interests are often developed through policy networks. The extent to which researchers are part of, or inform, policy networks is, therefore, an important aspect of research impact. Collaborations with policy advocacy groups, think tanks and government institutions can be listed as part of this description. In the LSHTM study, researchers emphasised the importance of establishing networks that extended beyond the formal bureaucracy as "knowledge users include policy-makers as well as the range of civil society organisations." [6]

### Political capital

Research impact on political capital takes into account the value of research evidence and researchers themselves in policy negotiations, in reaching in high quality agreements and improvements to the policy-making process, in the ability in societies to achieve agreed-upon ends, in the socio-political prestige resulting from innovations, and in increased national security [71]. For example, research on negotiation and consensus building methods on health issues could contribute to improving the quality of deliberations and reaching agreements that are reliable and satisfactory to the parties involved [72,73].

## III. Service impacts

The following categories help describe the impact of research on both health and intersectoral services.

• *Type of services: health/intersectoral*

• *Evidence-based practice*

• *Quality of care*

• *Information systems*

• *Services management*

• *Cost-containment and cost-effectiveness*

### Type of services

Health research, often primarily targeted at improving clinical and public health services, can have a range of impacts in this area that are detailed in the following sections on evidence based practice, quality of care, health information systems and on health services and systems management, including cost-effectiveness and cost-containment. Research on prevention and control measures for newly emerging diseases such as avian flu is another example of an area in which research can have an impact on health services [74].

In addition, health research can influence a range of other service sectors that contribute to public health. For example, in the LSHTM study researchers described how the WHO ethical guidelines, developed on the basis of a research project, for research on women and adolescents who were trafficked [43], were now being used to train journalists who conduct interviews on this topic and were also used in police training courses in several countries [6].

Living and work environments are associated with negative or positive effects on human health and are, therefore, critical areas for health research impact assessment [75]. For example, research on the health benefits of well-lit and safe places to work and exercise can contribute to reducing injury and promoting physical activity [76]. Impacts can also be described in the areas of measures undertaken, as a result of research, to reduce indoor and outdoor air pollution as well as in the use of more energy efficient devices [77].

Road traffic injuries are increasingly recognised as a major public health problem. Research could influence road safety and health, for example by highlighting issues for the regulation of vehicular use to facilitate safe walking and cycling, particularly for children [42] or by showing how the growth in sales of SUVs and pick-up trucks in the US contributed to a slower decline in road fatality rates there than in other industrialised countries [78].

Health research may also have an impact on vector control services, for example as related to studies on the cost-effectiveness of different types of insecticides to prevent malaria as well as on their safety for humans [39]. Additionally, clean drinking water and soil fertility are critically important to human health [79]. Thus, if applicable, research impact narratives could include descriptions of research impacts on food production and safety, water access and quality.

The impact of climate change and weather events on public health is another intersectoral area that is receiving increasing attention in health research. For example, in 2003, a heat wave resulted in numerous deaths across Europe, in France alone there were around 14,000 deaths [41]. This event raised the profile of climate change as a public health issue in Europe. Changes in forecasting and early warning systems as well as on related response mechanisms and guidelines, informed by research findings, are examples possible health research impacts in this area.

### Evidence-based practice

The use of research evidence by different groups involved in clinical diagnosis and decision-making is an indicator of research impact in health services [27]. Studies also indicate that in clinical decision-making and interviews between patients, their families and health professionals, the use of decision aids that are based on research evidence, can have significant effects on treatment decisions, choices and costs [80,81].

Research can also influence clinical practice and service delivery through [19,24,59]:

• *Adoption *of research findings and health technologies by health service providers

• *Adherence *to research-informed policies and guidelines and

• *Addressing barriers *to the use of research-informed interventions in the health system. Potential barriers include inflexible organisational workflows, inadequate resources and staff training as well as staff and patient attitudes and beliefs.

### Quality of care

Research-influenced changes in health services can lead to improved information on *quality of care *and improvements in quality of care itself. Quality of health care addresses [82]:

• *Efficacy *of health interventions. For example, one of the studies included in the LSHTM analysis was a clinical audit of tonsillectomy surgical techniques where the findings were that that 'hot' surgical techniques – diathermy or coblation – had a higher risk of complication than cold steel tonsillectomy methods [83].

• *Availability *of services, for example in different geographical regions.

• *Accessibility *of services, especially for disadvantaged and vulnerable individuals and groups.

• *Acceptability *of services provided, for example in terms of quality and cultural appropriateness.

• *Utilisation and coverage *of health services. Research can inform strategies to improve the coverage of health services and can also have impact through the development of methods to assess coverage. For example, availability and accessibility of services or interventions are often used as a proxy of health services coverage. However, it has been proposed that coverage should be assessed by estimating the probability that those who need a particular intervention will actually receive it, the probability of their requiring the intervention in the future and the probability of receiving effective health services based on previous experience [84].

• *Responsiveness *of health services to population health needs is a further area that can have impact vis-à-vis the quality of services.

### Information systems

A portion of health research is also directed toward improving the quality and efficiency of public health monitoring, reporting and evaluation. Possible indicators of the effects of research in this area include the accuracy, completeness, efficiency, relevance and timeliness of health monitoring and reporting systems, e.g. health information systems and GIS tools. For example, research in Turkey compared health information systems across 729 hospitals run by either the Ministry of Labor and Social Security or the Ministry of Health [85]. The study highlighted several differences in the use of health information systems across the two ministries that would need to be addressed given a proposed merger of the hospitals. This type of research could have an impact on the way health information systems are developed and used.

### Health systems and services management

The extent to which research contributes to changes in health systems management and administration is therefore another important area of potential impacts. For example, management of health services procurement and provisioning in both private and public sectors can also be influenced by research [86]. In assessing the impact of research on the management of health systems and markets, there is a broad range of considerations including health care financing and insurance. In addition, Hanson et al. [31] provide a conceptual framework to analyse non-financial constraints in health systems including sociopolitical changes, intersectoral issues, such as transport to health facilities and planning for long-term outcomes such as improving health by promoting female education.

### Cost-containment and cost-effectiveness

*Cost-containment*, while obviously related to health systems management is often a particular focus for assessing the impact of research on health services provision and health system management. This can have far-reaching implications for how health systems resources are allocated and used [87].

*Cost-effectiveness *of health services is another common focus in health research impact assessment and can be studied by analysing research-related changes in health systems in terms of both expenditure and related health outcomes [87]. In the LSHTM study, some research projects specifically included economic evaluations of the interventions being researched, for example the cost-effectiveness of social marketing of insecticide treated bed nets [88].

## IV. Societal impacts

Finally, research impacts can be described in terms of impacts at a societal level with reference to:

• *Knowledge, attitudes and behaviour*

• *Health literacy*

• *Health status*

• *Equity and human rights*

• *Macroeconomic/related to the economy*

• *Social capital and empowerment*

• *Culture and art*

• *Sustainable development outcomes*

### Knowledge, attitude and behaviour impacts

As a result of research dissemination and implementation strategies, various research impacts on knowledge, attitudes and behaviour could be expected or targeted. For example, research information could lead to changes in:

• Knowledge about and attitudes toward health risks and resources including the development of health self-efficacy. For example, in a study that was part of the LSHTM analysis, researchers had found that bilingual young people, while previously mainly viewed as an at risk group also served as a resource, often playing the role of translators and mediators in promoting their families' access to health care services [89]. These findings also led to changes in service institutions working with bilingual young people, to focus on both risks and resources in strategies aimed at improving access to health care [6].

• Healthy behaviours e.g. nutrition and diet, physical activity, safe sex, immunisations and improved hygiene practices can be promoted and adopted as a result of health research being disseminated, for example through health education and health promotion activities.

However, care must be taken in predicting or describing direct effects from research-based information being communicated and the desired effects occurring. There are a host of intermediary effects and influences that complicate that process, including culture, local and socio-historical contexts, the framing and relevance of the issues as well as perceived benefits and risks related to the research, so these should be noted if possible [29,90].

### Health literacy

Health literacy encompasses the wide range of skills and competencies required to find, understand, evaluate and use health information and concepts to make informed choices, reduce health risks and increase quality of life [91]. As such, health literacy is a key component of the complex relationship between knowledge, attitudes, decision-making, behaviour and health outcomes [30,92]. Thus, health literacy is a potential impact of health research. In addition health literacy can facilitate successful communication of health research or can be a potential roadblock in situations when health research is communicated in a manner not commensurate with the health literacy skills of the intended audience.

As one example of research that can have an impact on health literacy, traditionally malaria control strategies rely on the free distribution of insecticide treated bed nets through public health and donor agencies. However, research shows that approaches to malaria control approaches that raise awareness in communities about the causes and prevention of malaria and promote community skills and capacities to purchase and treat bed nets on their own, provide a more sustainable and equitable method of malaria control and significantly improve health outcomes [39,40].

### Health status

Research can contribute to improvements in health status by contributing to interventions that reduce morbidity, mortality and disability and promote health as well as by developing methods to measure and monitor health status. However, the attribution of health status effects to research can be challenging given the range of other influencing factors and there are ongoing efforts to address this challenge [18,28]. There are various measures for assessing health outcomes such as Quality Adjusted Life Years (QALYs); Disability Adjusted Life Years (DALYs); and Health Adjusted Life Expectancy (HALE) and a host of specific measures related to particular diseases/conditions. As is the case for assessment in general, the choice of measure usually reflects researchers' preferences, decision-makers' needs and the focus of assessment. Research can therefore also have impact by elucidating the values and assumptions underlying health status assessment measures so that these can be deliberated on by a broad range of stakeholders [93]. Research impact on health outcomes can thus be assessed both in terms of changes in health outcomes as well as in methods of measuring health outcomes.

### Equity and human rights

*Health equity *and the realisation of human rights are increasingly discussed as desired health research outcomes and need be taken into account at different levels of assessment, be they related to structure, process or outcome [94,95]. Research can also influence health equity outcomes by explicitly focusing on the needs of disadvantaged and vulnerable populations [96] as well as through the application of research to facilitate more equitable access to health care [67]. For example, there is concern that by focussing on mass interventions and targets set at the overall population level, the Millennium Development Goals may fail to address the needs of the most vulnerable and marginalised groups such as indigenous communities [97].

Human rights also include the right of individuals to participate in decisions that influence their lives and the right to information on the same. Additionally, the impact of participation on health outcomes is increasingly recognised. For example a randomised control trial showed that birth outcomes in a poor rural population in Nepal improved greatly through a low cost, potentially sustainable community-based participatory intervention with women's groups [98]. This type of research potentially has equity, human rights, methodological and health impacts.

### Macroeconomic/related to the economy

There are several areas in which the economic outcomes of health research can be assessed [87]. The term 'macroeconomic' is used here to distinguish between impact on the overall economy (though the scale of impact may vary) and impact in terms of cost-savings and cost-effectiveness in the health care system, which was described in an earlier section.

There are various ways in which health research can have impact on the economy. The extent to which individual researchers have access to this level of economic analysis may vary, but some multidisciplinary research collaborations explicitly include an economic analysis of the proposed research-based intervention or technology and others may rely on post hoc economic evaluations.

• Commercial outcomes can result from research in terms of monetary returns on investments in product development and marketing as well as from new or more efficient industrial processes [32,87].

• The impact of research on private markets, particularly with regard to the distribution of health interventions, needs to be further understood and assessed. For example, research evidence indicating that the distribution of soap to promote hygiene and nets to prevent malaria is more effective and as equitable when done through local markets than through the public health system, including to poor populations [38,99]. These findings have influenced how some public health programmes implement related strategies.

• Healthy workforce outcomes can also impact the economy. For example, analyses of the economic impact of health services research combine cost savings in health care provision with the economic value of a research intervention in terms of working days lost and produce a single metric of economic impact [100].

• The value of health gain (or loss) from research to societies in monetary terms. For example, the Funding First approach calculates the economic value of improved health and life expectancy resulting from investment in and application of research-based health interventions, e.g. to treat cardiovascular disease [28]. Evaluation of health outcomes in economic terms, however, is subject to criticism even from those who use the approach, as evaluating human life in monetary terms is ethically controversial. Other potential problems include the equity implications of such approaches, for instance, evaluating the impact of health research in terms of productivity could favour research primarily focused on those of working age and/or those with higher incomes [101].

### Social capital and empowerment

Social capital is increasingly viewed as a desired resource in societies and studies also find that there are links between social capital and health outcomes [4,5]. Therefore the contribution of research to the development of social capital and its assessment is a key area of impact. Social capital is measured through a variety of social research techniques and the context of the assessment is an important consideration, but many authors seem to agree on the importance of the following as measures of social capital [4,5]:

• Civic engagement and social cohesion (through social interaction, mobilisation and connections as well as the development of shared knowledge and concepts).

• Self-efficacy and collective efficacy (the ability and resources for problem solving at individual and societal levels).

• Trust (in other individuals and groups in society). For example, in a series of surveys in the UK on who people generally trusted to tell them the truth, doctors and teachers were listed first, scientists and the 'ordinary man/woman in the street' were near the middle and politicians and journalists were at the bottom of the list [102]. Similar surveys have been carried out in other countries [103]. Research can generate information that affects peoples' trust of different groups. In addition, trust in researchers themselves is associated with the support and use of science.

Research that empowers communities to access, understand and use health research informed interventions, as earlier discussed with reference to the malaria control example, and to take ownership of health promotion efforts [30] is another key area of health research impact.

### Culture and art

Culture refers to patterns of human learning and activity that lead to shared communication, artefacts and characteristics in societies; including language, patterns of behaviour, beliefs, identity, customs, traditions and other modes of expression [104]. These learned characteristics enable group members to hold and communicate shared meanings. The ability to influence culture is the also the ability to influence conceptions and categories that guide behaviour [105], including health behaviour. Health research generates new concepts and knowledge that influences societal culture (though the relationship works both ways). Health researchers are also constantly renegotiating the boundaries between what is considered traditional medicinal practice and research-based evidence as well as on what constitutes health and ill health. For instance, Maori researchers in New Zealand found that they were able to apply the methods and values of both indigenous and scientific knowledge in order to reach more comprehensive understandings of health and illness [106].

Arts and entertainment is a particular area where the influence of and on health research has generated interest in 'lay' society [107]. Some scientists serve as advisors on movies and TV shows. For example, the Journal of Public Health Policy reviewed the film *The Constant Gardener*. The film was based on John le Carre's book of the same title, in which the author mentions talking with Peter Godfrey-Faussett, a senior researcher at LSHTM, among others, in doing background research for the book. The review concludes with an observation of the potential impact of such work noting that,

This one production will be seen by millions of people around the world [and] will have done more to present the pharmaceutical industry's obstacles to improving health in developing countries ... than all health and science journals can in a year [108].

The influence of science on art and of art on science as well as on health and wellbeing certainly bears further research [109]. However, the interpretation and communication of science through art is increasingly considered an important means of science communication, not only in the mass media and movies, but also in museum collections on art and health as well as the art collections and programmes of major health research funders, such as the Wellcome Trust [110].

### Sustainable development

Sustainable development outcomes depend on a combination of several of the earlier described impacts. One commonly encountered conceptualization of sustainable development is that it enables all people throughout the world to satisfy their basic needs and enjoy a better quality of life without compromising the quality of life of future generations, for example as defined in the Rio Declaration on Environment and Development [111].

The UK was one of the first countries to establish a set of indicators to review sustainable development in 1996 and these have been reported on regularly and reviewed in annual reports [77]. Key elements of the UK sustainable development strategy are:

• Living within environmental limits

• Ensuring a strong, healthy and just society

• Achieving a sustainable economy

• Promoting good governance

• Using sound science responsibly.

Additionally, the continued ability to learn and adapt to new knowledge as it is produced is a key aspect of sustainable development. Thus, research may have a direct impact on sustainable development outcomes through the production of new knowledge on a range of topics, but can also help further develop concepts and assessment methods and help collect, validate and analyse data to guide sustainable development.

## Discussion

Researchers are often hard pressed to come up with coherent accounts of the significance and impact of their work as regularly required in a range of situations, from writing grant proposals to designing implementation strategies and in submissions to research assessment exercises. Increasingly health research funders expect measurable outcomes from their investment in research. While some may decry this as neglecting the inherent value of science, others point out to the underutilisation or misutilisation of health research or to the need to prioritise research funding based on identified societal needs [16-19]. There is also evidence that researchers' accounts of the impact of their work provide a reliable starting point for more in-depth analyses and can provide similar information to more specialised assessments, although the scope and level of analysis may vary [7].

In this light, we pragmatically set out to identify the areas where health research could be expected to have an impact and then organized these areas into a framework. The validity and usefulness of the Research Impact Framework was tested at the London School of Hygiene and Tropical Medicine. The framework categories were finalised based on feedback from researchers working on a wider range of health research topics as to which areas they thought provided a valid indication of the impact of their work and which categories they found most useful to describe the same.

While initially sceptical, LSHTM researchers found that using the Research Impact Framework prompted them to identify a wide range of impacts related to their work in a relatively systematic manner (compared to the ad hoc approaches they had previously used) [6]. Researchers' narratives contained specific and verifiable evidence and the standardized structure of the narratives facilitated analysis across projects that could inform research management, practice and assessment [6]. One caution we offer is to be aware of various biases that may influence identification and description of research impact. For instance there may be strong historical and institutional biases and incentives to identify only positive impacts from research. However, researchers interviewed in the LSHTM study were aware of potential negative impacts of their research, for example, stigma that could arise with the publication of research findings related to a particular group or community. We will continue to learn as much, if not more, about the relationship of science and society and research impacts by maintaining focus on scientific controversies as well as scientific developments.

## Conclusion

The Research Impact Framework provides a useful set of descriptive categories to help researchers identify and describe the impact of their work. It could also help researchers think through strategies to enhance the use of research-informed interventions as well as to identify unintended or harmful effects. The standardised structure of the framework also facilitates comparison of impact across projects, which is useful from an analytical and research management perspective. In addition, the framework should help health researchers systematically present the value of the work they have done as well as the work they wish to conduct in the future. This approach will further enable researchers to better explain what it is that they do and why they do it, not only to funders and governments, but also to review boards, colleagues, journalists and family and friends. This paper contributes to ongoing efforts seeking to understand, assess and explain the role, relevance and impact of health research.

## Competing interests

The author(s) declare that they have no competing interests.

## Authors' contributions

All the authors were involved in developing the Research Impact Framework described as well as in writing the paper and approving the final version. SK, NM and GW also applied and tested the framework in a study to analyse the impact of selected research projects at the London School of Hygiene and Tropical Medicine.

## Pre-publication history

The pre-publication history for this paper can be accessed here:



## Figures and Tables

**Table 1:** Guide to help structure research impact narratives

**Narrative areas**	**RESEARCH PROJECT/PROGRAMME DESCRIPTION**	**Key dates **(mm/yy)
**Description of research project/programme**	Topics/research area: Geopolitical contexts: Funders and budget: Research management, influencing events and challenges:	

**1. Research-related impact**	1.1 Type of problem/knowledge 1.2 Research methods used 1.3 Publications and papers 1.4 Products, patents and translatability potential 1.5 Research networks 1.6 Leadership and awards 1.7 Research management 1.8 Communication	

**2. Policy impact**	2.1 Level of policy-making 2.2 Type of policy 2.3 Nature of policy impact 2.4. Policy networks 2.5 Political capital	

**3. Service impact**	3.1 Type of services: health/intersectoral 3.2 Evidence-based practice 3.3 Quality of care 3.4 Information systems 3.5 Services management 3.6 Cost-containment and cost-effectiveness	

**4. Societal impact**	4.1 Knowledge, attitudes and behaviour 4.2 Health literacy 4.3 Health status 4.4 Equity and human rights 4.5 Macroeconomic/related to the economy 4.6 Social capital and empowerment 4.7 Culture and art 4.8 Sustainable development outcomes	

Note: The themes can be removed, added to, grouped, or modified as appropriate to the research being described and as relevant to funders' or research assessment criteria.

**Table 2:** Research Impact Framework

***Research-related impacts***	***Policy impacts***	***Service impacts***	***Societal impacts***
• Type of problem/knowledge • Research methods • Publications and papers • Products, patents and translatability potential • Research networks •Leadership and awards • Research management • Communication	• Level of policy-making • Type of policy • Nature of policy impact • Policy networks • Political capital	• Type of services: health/intersectoral • Evidence-based practice • Quality of care • Information systems • Services management • Cost-containment and cost-effectiveness	• Knowledge, attitudes and behaviour • Health literacy • Health status • Equity and human rights • Macroeconomic/related to the economy • Social capital and empowerment • Culture and art • Sustainable development outcomes
